# Evaluating lung cancer screening in China: Implications for eligibility criteria design from a microsimulation modeling approach

**DOI:** 10.1371/journal.pone.0173119

**Published:** 2017-03-08

**Authors:** Deirdre F. Sheehan, Steven D. Criss, G. Scott Gazelle, Pari V. Pandharipande, Chung Yin Kong

**Affiliations:** 1 Institute for Technology Assessment, Massachusetts General Hospital, Boston, Massachusetts, United States of America; 2 Harvard Medical School, Boston, Massachusetts, United States of America; 3 Harvard T.H. Chan School of Public Health, Boston, Massachusetts, United States of America; University of Groningen, University Medical Center Groningen, NETHERLANDS

## Abstract

More than half of males in China are current smokers and evidence from western countries tells us that an unprecedented number of smoking-attributable deaths will occur as the Chinese population ages. We used the China Lung Cancer Policy Model (LCPM) to simulate effects of computed tomography (CT)-based lung cancer screening in China, comparing the impact of a screening guideline published in 2015 by a Chinese expert group to a version developed for the United States by the U.S. Centers for Medicare & Medicaid Services (CMS). The China LCPM, built using an existing lung cancer microsimulation model, can project population outcomes associated with interventions for smoking-related diseases. After calibrating the model to published Chinese smoking prevalence and lung cancer mortality rates, we simulated screening from 2016 to 2050 based on eligibility criteria from the CMS and Chinese guidelines, which differ by age to begin and end screening, pack-years smoked, and years since quitting. Outcomes included number of screens, mortality reduction, and life-years saved for each strategy. We projected that in the absence of screening, 14.98 million lung cancer deaths would occur between 2016 and 2050. Screening with the CMS guideline would prevent 0.72 million deaths and 5.8 million life-years lost, resulting in 6.58% and 1.97% mortality reduction in males and females, respectively. Screening with the Chinese guideline would prevent 0.74 million deaths and 6.6 million life-years lost, resulting in 6.30% and 2.79% mortality reduction in males and females, respectively. Through 2050, 1.43 billion screens would be required using the Chinese screening strategy, compared to 988 million screens using the CMS guideline. In conclusion, CT-based lung cancer screening implemented in 2016 and based on the Chinese screening guideline would prevent about 20,000 (2.9%) more lung cancer deaths through 2050, but would require about 445 million (44.7%) more screens than the CMS guideline.

## Introduction

China is the largest consumer of tobacco in the world, with over 300 million current smokers.[1] This figure is mainly driven by adult males: over half of Chinese men are estimated to be current smokers, compared to under 3% of women.[1] Although smoking prevalence in China has been slowly declining, the male smoking prevalence remains above 50% and is projected to be over 40% in the year 2050.[2] The age-adjusted relative risk of death is significantly higher for smokers than for lifelong non-smokers, even among smokers with very few pack-years (a measure that incorporates both cigarette packs smoked per day and years of being a smoker—i.e. one pack-year is equal to one pack of cigarettes smoked per day for one year, or 0.5 packs of cigarettes per day for 2 years, etc.).[3] Among males in China, lung cancer has the highest incidence rate of all major cancers and among females it is second only to breast cancer.[4, 5] According to data from the World Health Organization, an estimated 422,000 males and 175,000 females in China died of lung cancer in 2012 alone.[6] The Chinese population’s demonstrated high smoking prevalence will inevitably result in millions of smoking-attributable deaths in the coming decades, many of which will be caused by lung cancer.[2]

Screening-based policies can potentially have a large impact on the smoking-related disease burden in future decades. Lung cancer screening with computed tomography (CT) for current and former heavy smokers was estimated to result in a 20% mortality reduction among screened individuals compared to chest x-ray in the National Lung Screening Trial.[7] These findings led to recommendations in 2015 from the U.S. Centers for Medicare & Medicaid Services (CMS) that all current and former smokers ages 55–77 with a smoking history of at least 30 pack-years and no more than 15 years since quitting be screened for lung cancer with CT annually.[8]

In 2015, experts appointed by the Chinese National Health and Family Planning Commission issued revised national lung cancer CT screening guidelines for China that differed substantially from CMS screening eligibility criteria. Chinese guidelines recommend annual screening CT exams for current and former smokers ages 50–74 with a smoking history of 20 pack-years and no more than five years since quitting.[9] China has initiated several large-scale lung cancer screening programs, such as the demonstration program of lung cancer screening with CT launched in 2010 for high-risk populations at multiple study sites, which aims to determine the feasibility of conducting population-based CT screening exams in China.[10] Additionally, the Cancer Screening Project in Urban Areas of China, initiated by the Ministry of Health in 2012, will provide an estimated 210,000 individuals with free lung cancer screenings between 2012 and 2017.[11]

The purpose of this study was to use the China Lung Cancer Policy Model (LCPM), a computer-based simulation model, to estimate mortality reduction through 2050 if CT-based lung cancer screening were implemented throughout China in 2016, with screening eligibility criteria based on CMS or Chinese guidelines. In addition, we estimated the number of screens that would be required for each strategy to achieve this mortality reduction.

## Methods

### Overview of the Lung Cancer Policy Model

Microsimulation is a popular modeling method for health economic and clinical evaluations that simulates hypothetical individuals to estimate health outcomes.[12] Results of analyses using microsimulation methods have been used by the U.S. Preventive Services Task Force (USPSTF) for breast,[13] colorectal,[14] and lung cancer[15] screening guidelines, and by the CMS. [16] The China LCPM is built on a well-established microsimulation model that simulates an individual patient’s lung cancer development, progression, detection, follow-up, and treatment, as well as patient survival.[17–19] The LCPM has been used to evaluate the impact of tobacco control strategies on mortality reduction and the cost-effectiveness of imaging-based screening programs for lung cancer.[20–22] In the model, both a “true” and observed disease stage are assigned to individuals in the population, the former being based on the individual’s simulated disease characteristics (tumor size, nodal involvement, and distant spread) and the latter being based on the individual’s true disease characteristics, presence of any benign pulmonary nodules, and results from any diagnostic or staging tests.[19] A pulmonary nodule suspicious for lung cancer may be detected subsequent to symptoms, detected by incidental detection during an imaging exam given for reasons unrelated to lung cancer (e.g., trauma), or detected on a screening examination.[19] Observed and true stages may not match if a cancer is undiagnosed or mis-staged by a test result.[19] A detailed description of the LCPM is publicly available, as recorded within a designated National Cancer Institute website (http://www.cisnet.cancer.gov/lung/profiles.html).

The LCPM was originally created to project population-level lung cancer outcomes in the United States; adaptations were made to the LCPM to fit the Chinese population. Model inputs (Table 1) included smoking intensity, smoking initiation rates, smoking cessation rates, and rates of mortality from causes other than lung cancer, which were based on published literature.[2, 23–26]

**Table 1:** China-specific input parameters and calibration targets of the Lung Cancer Policy Model.

	Definition	Values	Source
Smoking prevalence	Proportion of the adult population (age >15) who currently smoke cigarettes	**Male**—1991: 0.66; 1993: 0.65; 1997: 0.62; 2000: 0.58; 2004: 0.59; 2006: 0.57 **Female**—1991: 0.062; 1993: 0.061; 1997: 0.055; 2000: 0.053; 2004: 0.041; 2006: 0.035	China Health and Nutrition Survey
Smoking cessation rate	Proportion of current smokers who quit smoking cigarettes each year	**Male**—Age 30–65: 0.02; Age >65: 0.03 **Female—**Age >35: 0.02	Levy et al 2014
Cigarettes per day	Cigarettes smoked per day by current smokers	**Male by age in 2006** (Chen, average of urban and rural)—Age 37: 16.56; Age 46: 17.44; Age 55: 15.76; Age 66: 13.2; Age 74: 12.2 **Male and female by year** (Ng)—1980: 15.5 (13.9, 17.1); 1996: 18.2 (17.3, 19.1); 2006: 21.8 (20.6, 23.0); 2012: 22.3 (20.7, 24.4) **Male by year** (Qian, calculated)—1998: 15.8; 2003: 21.3 **Female by year** (Qian, calculated)—1998: 12.3; 2003: 15.8	Chen et al 2015; Ng et al 2015; Qian et al 2010
Other-cause mortality	Mortality rate for causes other than lung cancer	Individualized based on age and sex	Global Burden of Disease, 1990 and 2010
Lung cancer mortality	Number of lung cancer deaths	**Male**—422,000 **Female**—175,000	GLOBOCAN 2012
Lung cancer histology distribution	Percent of total lung cancer cases diagnosed as each histological subtype	**Male**—1991–95: Adenocarcinoma 30%, Squamous cell 54%, Small cell 13%, Large cell/Other 3%; 1996–2000: Adenocarcinoma 31%, Squamous cell 52%, Small cell 13%, Large cell/Other 4%; 2001–05: Adenocarcinoma 33%, Squamous cell 52%, Small cell 10%, Large cell/Other 5% **Female**—1995–95: Adenocarcinoma 51%, Squamous cell 32%, Small cell 15%, Large cell/Other 2%; 1996–2000: Adenocarcinoma 55%, Squamous cell 32%, Small cell 10%, Large cell/Other 3%; 2001–05: Adenocarcinoma 54%, Squamous cell 34%, Small cell 8%, Large cell/Other 4%	Kong et al 2014

Chinese smoking prevalence was calibrated to rates published by the China Health and Nutrition Survey (CHNS). The CHNS, a periodic survey that took place over three-day periods in nine Chinese provinces, documented current smoking rates for men and women ages 35–74 during the years 1991–2006, covering about 4,400 households and 19,000 individuals selected using a weighted sampling scheme within provinces.[27] Smoking cessation rates vary by age and gender, and are tracked from age 30 for males and age 35 for females.[2] The cigarettes per day input parameters are based on numerous sources reporting on smoking intensity by age, gender, and calendar year.[23, 25, 26] Other-cause mortality in China was incorporated into the model using estimates from the Global Burden of Disease, which provides estimates for all-cause and lung cancer-specific mortality for all age groups in 1990 and 2010.[24] Other-cause mortality risks in the LCPM are stratified by age, sex, and smoking intensity. Lung cancer mortality rates were calibrated to data from the International Agency for Research on Cancer (IARC) GLOBOCAN project, which provides Chinese cancer mortality statistics extracted from the World Health Organization database for years between 1987 and 2012.[6] Lung cancer histology distribution was calibrated to data from a population-based study in China reporting on incidence of lung cancer by histological type during 1981–2005.[28]

### Screening strategies

Health effects of multiple screening scenarios were evaluated using the calibrated model. The base case scenario was no lung cancer screening. The base case scenario was compared to scenarios that implemented lung cancer screening criteria based on CMS or Chinese guidelines.[8, 9] In the scenario based on CMS screening criteria, current and former smokers ages 55–77 with at least 30 pack-years of smoking history and fewer than 15 years since quitting were screened for lung cancer annually with CT. The corresponding values for Chinese screening criteria were ages 50–74, at least 20 pack-years and fewer than five years since quitting. See S1 Table for comparison of eligibility criteria. In these scenarios, CT screening was fully implemented in the year 2016 and continued to the year 2050. We assumed 100% screening adherence to estimate the full potential benefits and harms of screening. The effects of screening adherence rate and smoking cessation rate are addressed in the sensitivity analysis.

### Model simulations

Each model run simulated a population of people ages 15 to 84 during years 1975–2050, beginning with two million men and two million women at age 15 from each birth cohort, and projected outcomes for current, former, and never smokers depending on screening strategy. Results from the simulations were scaled to estimates for the actual Chinese population from the U.S. Census Bureau international database, which projects population by age through 2050.[29] For each scenario, the China LCPM outputs include lung cancer incidence, lung cancer mortality, number of screening CT exams, and total population size, for both men and women, stratified by smoking status.

### Model outcomes

Deaths from each smoking category—current, former, or never smokers—were summed by calendar year to determine the total number of projected lung cancer deaths each year within the Chinese population. Lung cancer mortality reduction for each strategy was calculated by subtracting the total lung cancer mortality of that strategy during the years 2016 to 2050 from the total baseline (no screening) lung cancer mortality during these years, then dividing by the baseline mortality. A similar calculation was used to calculate life-years saved. To calculate percent of the population eligible for screening each year from 2016 to 2050, the total number of screening CT exams each year was divided by the adult population each year.

### Sensitivity analysis

Sensitivity analyses were conducted to determine the extent to which the model’s outputs would change if adherence to the recommended screening strategy was not 100% in the Chinese population and if the smoking cessation rate base case value was halved or doubled.

## Results

### Model calibration

**Fig 1:**
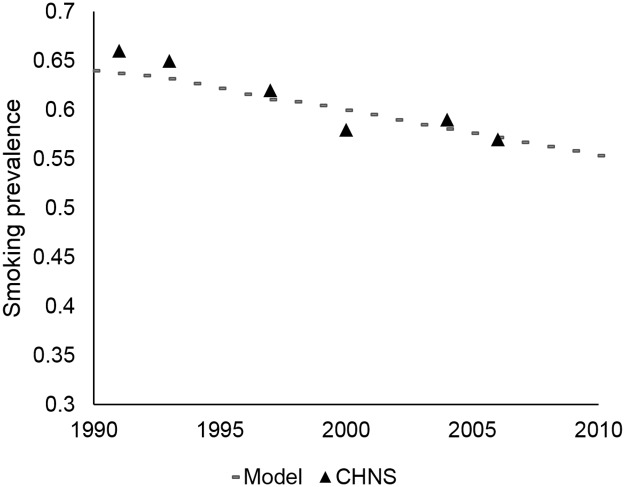
Smoking prevalence calibration, males. Model smoking prevalence calibrated to data from the China Health and Nutrition Survey (CHNS).

**Fig 2:**
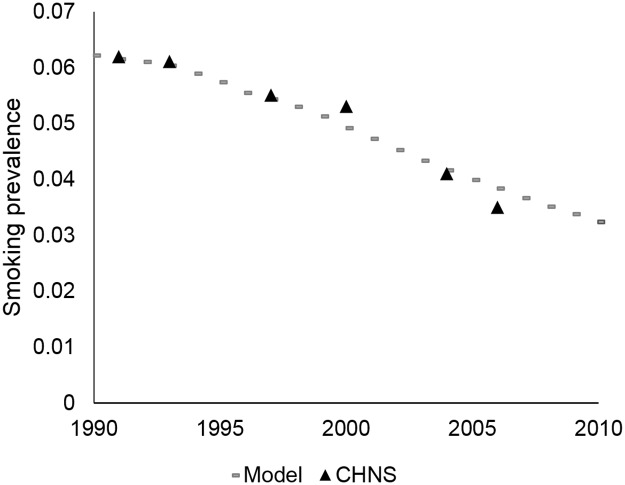
Smoking prevalence calibration, females. Model smoking prevalence calibrated to data from the China Health and Nutrition Survey (CHNS).

**Fig 3:**
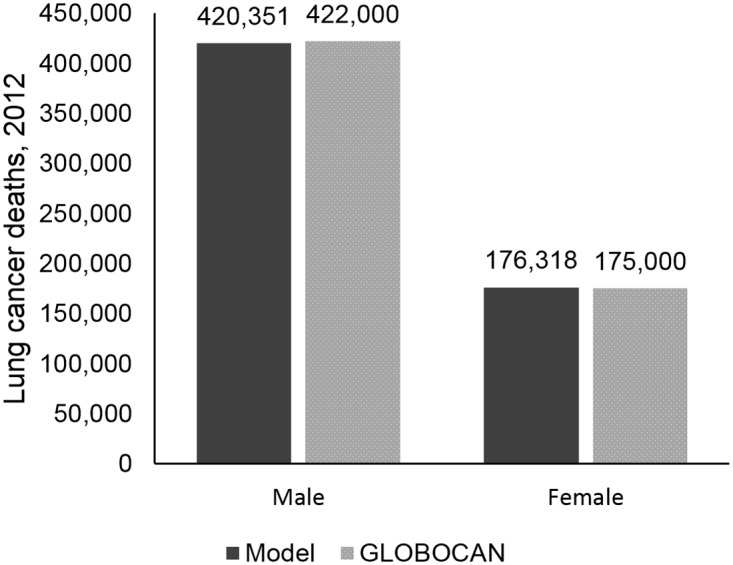
Lung cancer mortality calibration. Model output and calibration targets from GLOBOCAN.

We calibrated smoking prevalence to data from the China Health and Nutrition Survey (Figs 1 and 2) and lung cancer mortality to GLOBOCAN 2012 data (Fig 3).[6] The LCPM estimated 420,351 lung cancer deaths for males in 2012, compared to 422,000 estimated by GLOBOCAN, and 176,318 lung cancer deaths for females in 2012, compared to GLOBOCAN’s estimate of 175,000. Model results for lung cancer histology distribution are compared to calibration targets in S1 Fig.

### Mortality in the absence of screening

We projected that, in the absence of screening, 14,985,127 lung cancer deaths would occur in China between 2016 and 2050 (9,257,019 in males and 5,728,108 in females). Among males, the majority of these deaths would be among current smokers (5,537,917) and former smokers (3,572,169), with far fewer deaths among never-smokers (146,933). Among females, the majority of deaths would occur among never-smokers (3,351,513), followed by current smokers (1,603,169) and former smokers (773,426).

### Eligibility for screening

**Fig 4:**
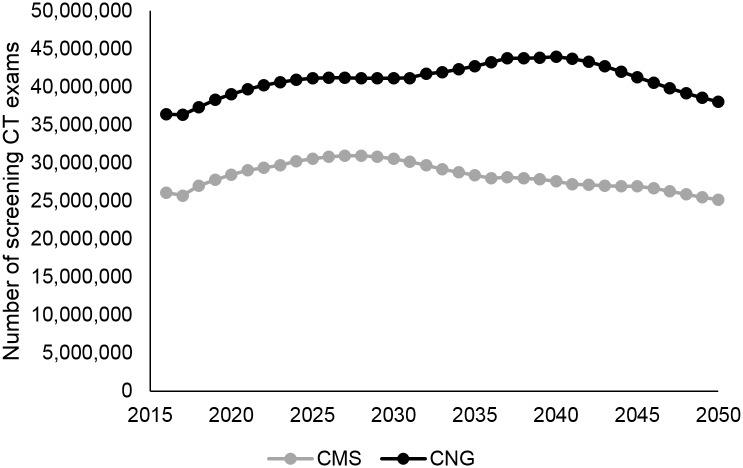
Number of people eligible for screening, based on the Centers for Medicare & Medicaid Services (CMS) or Chinese National Guideline (CNG) criteria.

Through the year 2050, about 988 million people would be eligible for screening based on CMS guidelines, compared to about 1.43 billion people based on Chinese screening guidelines. Fig 4 shows eligibility for lung cancer screening by year for each of the two screening guidelines. From 2016 to 2050, a consistently larger portion of the population would be eligible for lung cancer screening based on Chinese guidelines compared to the portion eligible under CMS guidelines.

### Mortality reduction by screening strategy

**Fig 5:**
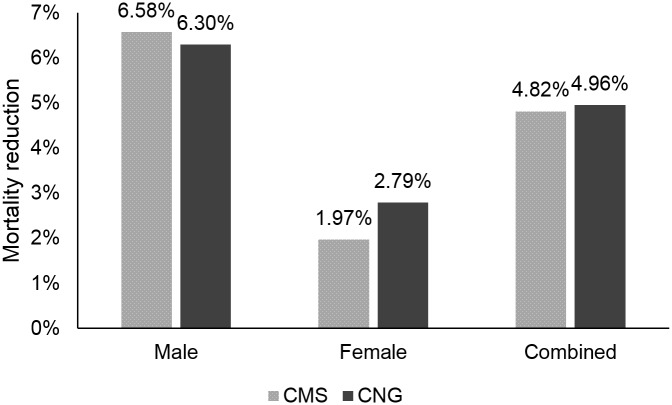
Cumulative lung cancer-specific mortality reduction, 2016–2050. Percent mortality reduction predicted by the Lung Cancer Policy Model is shown for males and females, both separately and combined, following screening with Centers for Medicare & Medicaid Services (CMS) criteria and screening with Chinese national guideline (CNG) criteria.

**Fig 6:**
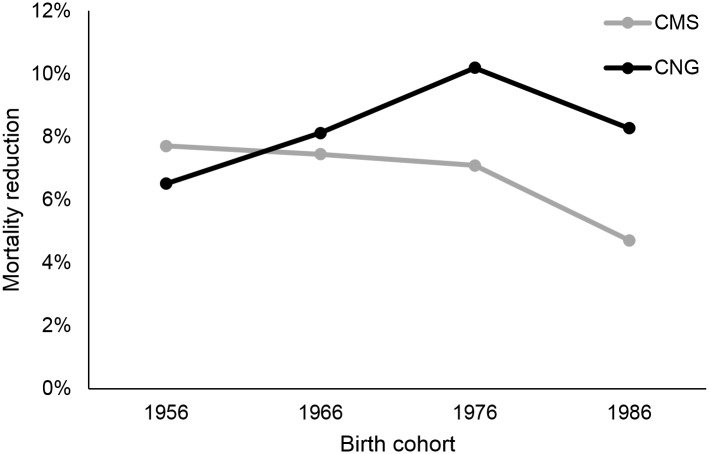
Mortality reduction by birth cohort for males.

**Fig 7:**
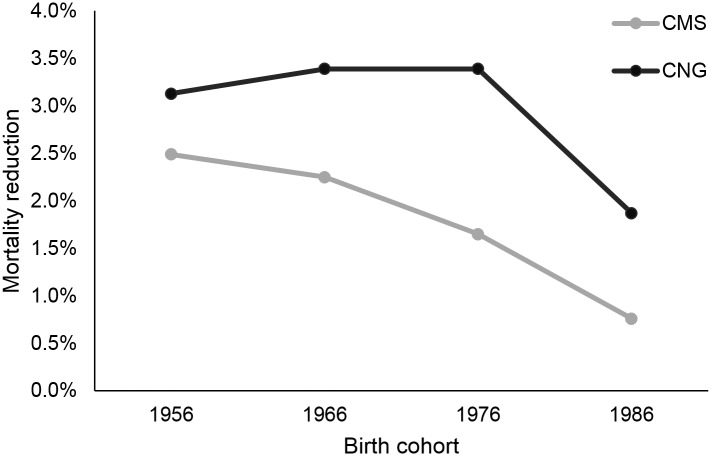
Mortality reduction by birth cohort for females.

By 2050, our model predicted that lung cancer screening with CMS guidelines would prevent 721,589 deaths, while screening with Chinese guidelines would prevent 742,563 deaths. Fig 5 shows cumulative lung cancer-specific mortality reduction as a result of each strategy. In males, screening based on CMS criteria results in higher lung cancer-specific mortality reduction (6.58%) than screening based on Chinese criteria (6.30%). Conversely, in females, Chinese screening criteria results in higher mortality reduction (2.79%) than CMS criteria (1.97%). For the CMS strategy, mortality reduction is higher in older male cohorts than younger male cohorts, and the opposite is seen for the Chinese strategy (Figs 6 and 7). Cumulative mortality reduction by strategy as a function of calendar year begins to converge as the year approaches 2050 for both male and female (S2 Fig). Lung cancer deaths projected for each screening strategy, stratified by smoking status, are shown in Table 2. Lung cancer screening eligibility that is based on personal smoking history, which is the case in both strategies considered, does not affect mortality in never-smokers.

**Table 2:** Number of lung cancer deaths (% of lung cancer deaths) among current, former, and never smokers for male and female through 2050 following implementation of screening in 2016 with CMS or Chinese National Guideline (CNG) eligibility.

	CMS	CNG
**Male**		
Current	5,121,750 (59.2)	5,064,225 (58.4)
Former	3,379,508 (39.1)	3,462,941 (39.9)
Never	146,933 (1.7)	146,933 (1.7)
**Female**		
Current	1,516,785 (27.0)	1,467,832 (26.4)
Former	747,049 (13.3)	749,120 (13.4)
Never	3,351,513 (59.7)	3,351,513 (60.2)

Because the size of the population being screened differs between screening strategies, mortality reduction percentages are calculated using the total simulated population for a fair comparison. Mortality reduction calculated within the subgroup of the population eligible for screening in each strategy is notably higher (S3 Fig). Within the subgroup of the population who met CMS eligibility criteria, the mortality reduction projection was 19.7% for the 1960 birth cohort. This estimate is similar to the 20.0% mortality reduction finding from the National Lung Screening Trial and provides a source of validation for our results. [7]

The CMS strategy would require 1,525 and 426 screening CT exams per death prevented for males and females, respectively. The ratio of screens to deaths averted would be higher for the Chinese strategy, with 2,302 and 574 screening CT exams per death prevented for males and females, respectively.

### Life-years saved

Lung cancer screening with CMS guidelines would save an estimated 5,798,331 life-years through 2050, resulting in 8.0 life-years saved per lung cancer death prevented. Screening with Chinese guidelines would save an estimated 6,623,148 life-years during this time period, resulting in 8.9 life-years saved per lung cancer death prevented.

### Sensitivity analysis

Sensitivity analyses determined the extent to which the outputs would change if adherence to screening in the population were not 100% (S2 Table, S4 Fig). Additional sensitivity analyses determined the impact of changes in smoking cessation that occur in the model at the start of screening (S3 Table).

## Discussion

With over 300 million current smokers—most of whom are men—China has the highest number of current smokers in the world.[1] Lung cancer incidence resulting from the persistently high smoking prevalence in China will undoubtedly be large, but widespread CT-based lung cancer screening for high-risk current and former smokers has the potential to attenuate mortality rates. The China LCPM simulated smoking prevalence and subsequent lung cancer mortality through 2050, imposing two different screening guidelines on the simulated population. Results show that the Chinese guidelines would prevent more deaths (742,563) than the CMS guidelines (721,589), but would also require many more screens (1.43 billion) than the CMS guidelines (988 million). The number of deaths prevented translates to 6.58% mortality reduction for the CMS strategy and 6.30% mortality reduction for the Chinese strategy among males and 1.97% and 2.79%, respectively, for females. Considering a screening burden well over one billion screening CT exams through 2050, the resulting mortality reduction of the Chinese guideline appears relatively small.

Some of the observed differences between the two screening strategies considered may be explained by the fact that Chinese guidelines include younger and lighter smokers, as well as former smokers who quit smoking more recently. For example, female mortality reduction is projected to be about 50% higher with the Chinese strategy because female smokers in China tend to be lighter smokers than males. These female lighter smokers are not being captured by the CMS strategy, which requires a smoking history of at least 30 pack-years. Secondly, life-years saved through 2050 are projected to be about 14% higher with the Chinese strategy because younger smokers with fewer pack-years are screened, and this population has a longer life-expectancy, on average, at the start of screening. Thirdly, about 45% more people are eligible for screening in the Chinese strategy because more light and former smokers that are excluded by the CMS eligibility criteria qualify for screening.

As the CMS screening criteria capture older, heavier smokers, the mortality reduction in males for the CMS strategy is driven by the older birth cohorts, whereas the opposite is true for the Chinese strategy (Fig 6). Cumulative mortality reduction in males begins to converge as the year approaches 2050 because older males with heavier smoking histories are being replaced by younger males with lighter smoking histories in the Chinese population.

Mortality reduction and life-years saved for each strategy were simulated assuming complete screening adherence for the eligible population. Sensitivity analyses assuming imperfect adherence to screening project that the number of screening CT exams will decline linearly with declining population adherence. Additionally, sensitivity analyses confirm that large changes in smoking cessation rates (base case cessation rates doubled or halved beginning at the start of screening) are not projected to have a notable impact on mortality reduction through 2050. This can be explained by the fact that the base case smoking cessation rate is small to begin with (2–3% per year), and that much of the population will have already established smoking histories that meet the screening eligibility criteria prior to quitting.

Lung cancer deaths among never-smokers would not be affected by screening if eligibility criteria includes only ever-smokers, which is the case in both strategies considered. Never-smokers account for 23.3% of the total lung cancer mortality through 2050 in the absence of screening, driven mainly by deaths among never-smoking females. The model predicted 3,351,513 lung cancer deaths through 2050 among never-smoking females in the absence of screening, which translates to 58.5% of the lung cancer deaths among females. The proportion of total female lung cancer deaths occupied by never-smokers is projected to be even higher with either of the two screening strategies considered. Although smoking prevalence among females is under 3%,[1] the high burden of lung cancer in female never-smokers can be explained by regular exposure to second-hand smoke and indoor air pollution, caused at least in part by indoor cooking in China.[30]. There have been some international studies published on lung cancer screening that include never-smokers.[31, 32] A published study assessing the effects of lung cancer screening for never-smokers predicted that never-smokers with high risks of lung cancer relative to those at average risk (relative risks of 15 to 35) may have similar to better trade-offs between benefits and harms of screening compared to smokers recommended for screening by the USPSTF guidelines, but that screening would not be beneficial for most never-smokers.[33] However, the actual relative risk of lung cancer for never-smokers exposed to high air pollution levels in China remains unknown. Research has also shown an association between exposure to secondhand tobacco smoke and disease such as coronary artery calcification in never-smokers.[34] Chinese screening guidelines that differ between males and females were not within the scope of our current study, but they may be reasonable to consider in the future, especially considering the large proportion of female lung cancer deaths occurring among never-smokers. Additionally, some studies have shown that risk-based screening can provide better mortality reduction, so such a screening approach may be logical to consider in China.[35–39]

Our model-based findings highlight the shortcomings of CT-based lung cancer screening as a mortality-reduction strategy. A study published in 2014 by Levy et al. simulated future Chinese smoking prevalence and smoking attributable deaths through 2050 following the implementation of various tobacco control policies.[2] Our model projected that screening with CT would avert more premature deaths than would certain interventions such as strong health warnings and youth tobacco access enforcement. However, it projected saving many fewer lives than would interventions such as comprehensive smoke-free air laws, a tax on cigarettes at 75% of retail price, and cessation treatment policies. The figures on premature deaths averted by the two studies cannot be directly compared—Levy et al. reported on all smoking attributable deaths, while our study reports lung cancer deaths—but based on these results, the long-term overall number of deaths averted would likely be much higher from strengthened tobacco control programs than from lung cancer screening programs.

### Limitations

This analysis has several limitations. China has notoriously high air pollution levels and the particulate matter in outdoor air pollution is associated with increased risk of lung cancer.[40] Indoor air pollution, which is created by biomass fuel and coal used for cooking and heating, is strongly associated with lung cancer in non-smoking women in China.[30] These trends in air pollution and their effects on lung cancer were not incorporated into the China LCPM. However, baseline lung cancer mortality was calibrated to published values for the Chinese population, therefore implicitly accounting for lung cancer due to pollution at baseline. Additionally, the model does not consider differences in smoking prevalence between rural and urban areas of China. Finally, the inputs used for cigarettes per day are based on data limited to the years 1980–2012, which affects who is eligible for screening.

The Chinese guidelines state that annual screening is also recommended for people living in regions with “high lung cancer incidence caused by specific environmental or occupational carcinogens,” though more detailed guidelines for people in these areas are not specified.[9] Screening based on environmental or occupational exposure was not incorporated into our current model. Many people in China, especially women, are non-smokers, but are still at an increased risk for lung cancer due to heavy exposure to environmental tobacco smoke at work.[30] Screening eligibility based on Chinese guidelines that incorporate environmental or occupational exposure would likely save more lives and require more screening tests than the CMS guidelines. To quantify the impact of this additional eligibility criteria, the guidelines would need to be more specific.

The projections used for the Chinese population through 2050 were based on the U.S. Census Bureau International database, and were created prior to the end of China’s one-child policy in December 2015. Accordingly, population estimates for birth cohorts 2016 and onward will likely be underestimates. This will not affect our model until 2031, when the 2016 birth cohort will reach age 15 and enter into our model.

## Conclusion

In China, CT-based lung cancer screening implemented in 2016 and based on the Chinese screening guideline would prevent about 20,000 (2.9%) more lung cancer deaths through 2050 compared to the CMS guideline, but implementation would require about 445 million (44.7%) more screens. Number of lives saved per number of screens performed will depend largely on screening eligibility criteria. Based on this analysis, it appears that screening will have a small (<7%) impact on mortality reduction if based on current Chinese or U.S. CMS guidelines.

## Supporting information

S1 Table Centers for Medicare and Medicaid Services (CMS) lung cancer screening eligibility criteria vs. Chinese National Guidelines (CNG) lung cancer screening eligibility criteria. (PDF)

S2 Table Sensitivity analysis: Mortality reduction and number of screens 2016 to 2050 with varying screening adherence for males and females, using CMS screening eligibility criteria. (PDF)

S3 Table Sensitivity analysis: Mortality reduction and number of screens 2016 to 2050 with varying smoking cessation for males and females, comparing CMS and Chinese National Guidelines (CNG) screening eligibility criteria. (PDF)

S1 Fig Lung cancer histology distribution calibration: model output versus calibration targets. (PDF)

S2 Fig Cumulative lung cancer mortality reduction as a function of calendar year, compared to no screening. (PDF)

S3 Fig Mortality reduction compared to National Lung Screening Trial (NLST) for the 1960 birth cohort. (PDF)

S4 Fig Number of screening CT exams based on screening adherence rates to CMS strategy, 2016 to 2050. (PDF)

S5 Fig Cigarettes per day, males. (PDF)
