# Supplementary material for: Evaluating lung cancer screening in China: Implications for eligibility criteria design from a microsimulation modeling approach
Source: PLoS One. 2017 Mar 8;12(3):e0173119. doi: 10.1371/journal.pone.0173119 (PMC5342219; doi:10.1371/journal.pone.0173119)
Supplement: S1 Table — (PDF) [file pone.0173119.s001.pdf]

S1 Table. Centers for Medicare and Medicaid Services (CMS) lung cancer screening eligibility criteria vs. Chinese national guidelines (CNG) lung cancer screening eligibility criteria.

	CMS	CNG
Age start screening	55	50
Age stop screening	77	74
Minimum pack-years smoked	30	20
Maximum years since quit smoking	15	5
