# Supplementary material for: Evaluating lung cancer screening in China: Implications for eligibility criteria design from a microsimulation modeling approach
Source: PLoS One. 2017 Mar 8;12(3):e0173119. doi: 10.1371/journal.pone.0173119 (PMC5342219; doi:10.1371/journal.pone.0173119)
Supplement: S2 Table — (PDF) [file pone.0173119.s002.pdf]

S2 Table. Sensitivity analysis: Mortality reduction and number of screens 2016 to 2050 with varying screening adherence for males and females, using CMS screening eligibility criteria.

	100% adherence	80% adherence	60% adherence	40% adherence	20% adherence
**Males**					
% mortality reduction	6.58	4.98	3.68	2.37	1.09
Number of screens	940,402,571	733,964,644	538,121,966	336,112,494	141,498,868
**Females**					
% mortality reduction	1.97	1.51	1.14	0.73	0.32
Number of screens	48,040,189	37,213,339	27,230,654	17,016,651	7,097,013
