# Supplementary material for: Evaluating lung cancer screening in China: Implications for eligibility criteria design from a microsimulation modeling approach
Source: PLoS One. 2017 Mar 8;12(3):e0173119. doi: 10.1371/journal.pone.0173119 (PMC5342219; doi:10.1371/journal.pone.0173119)
Supplement: S3 Table — (PDF) [file pone.0173119.s003.pdf]

S3 Table. Sensitivity analysis: Mortality reduction and number of screens 2016 to 2050 with varying smoking cessation for males and females, comparing CMS and Chinese national guidelines (CNG) screening eligibility criteria.

	Original cessation	Doubled cessation	Halved cessation
**Males**			
**CMS**			
Mortality reduction	6.577%	6.569%	6.583%
Number of screens	940,402,571	884,016,491	977,425,121
**CNG**			
Mortality reduction	6.297%	6.174%	6.390%
Number of screens	1,342,089,617	1,196,905,085	1,442,988,814
**Females**			
**CMS**			
Mortality reduction	1.969%	1.947%	1.982%
Number of screens	48,040,189	46,361,076	49,097,831
**CNG**			
Mortality reduction	2.787%	2.722%	2.830%
Number of screens	91,589,565	86,408,251	95,162,942
