# Supplementary material for: Evaluating lung cancer screening in China: Implications for eligibility criteria design from a microsimulation modeling approach
Source: PLoS One. 2017 Mar 8;12(3):e0173119. doi: 10.1371/journal.pone.0173119 (PMC5342219; doi:10.1371/journal.pone.0173119)

S1 Fig. Lung cancer histology distribution calibration: model output versus calibration targets. A) Male B) Female.

A)

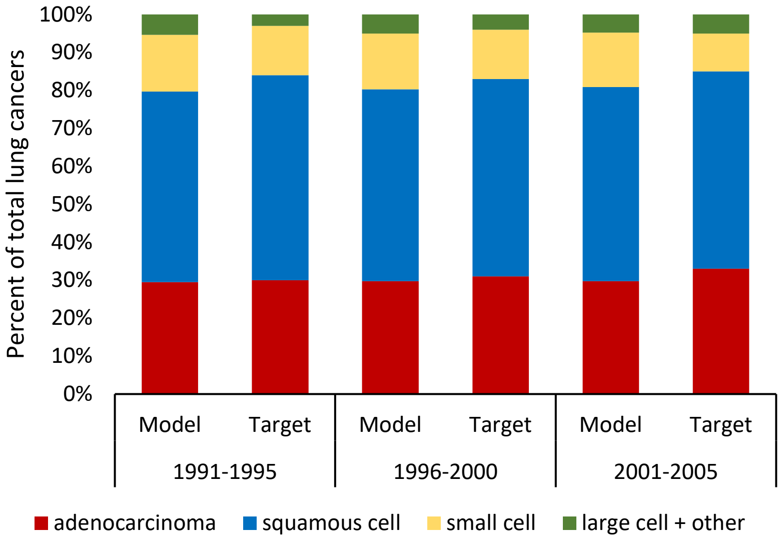

B)

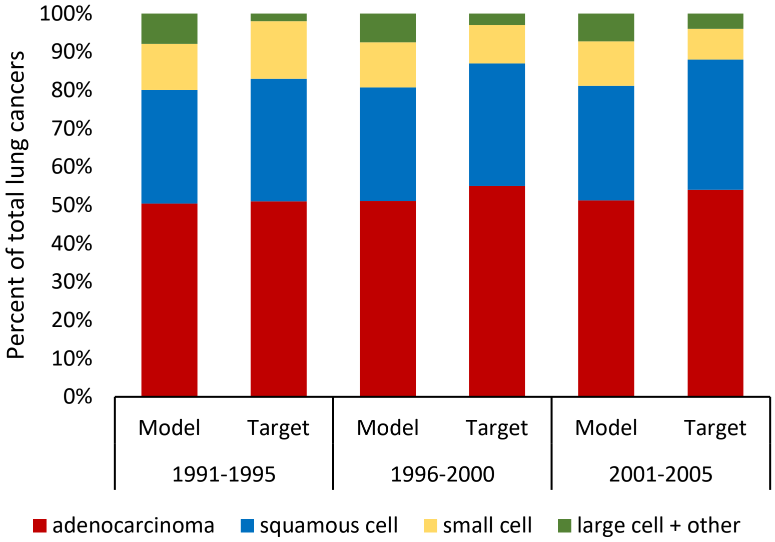

Supplement: S1 Fig — (PDF) [file pone.0173119.s004.pdf]
