# Supplementary material for: Evaluating lung cancer screening in China: Implications for eligibility criteria design from a microsimulation modeling approach
Source: PLoS One. 2017 Mar 8;12(3):e0173119. doi: 10.1371/journal.pone.0173119 (PMC5342219; doi:10.1371/journal.pone.0173119)

S2 Fig. Cumulative lung cancer mortality reduction as a function of calendar year, compared to no screening. A) Males B) Females.

A)

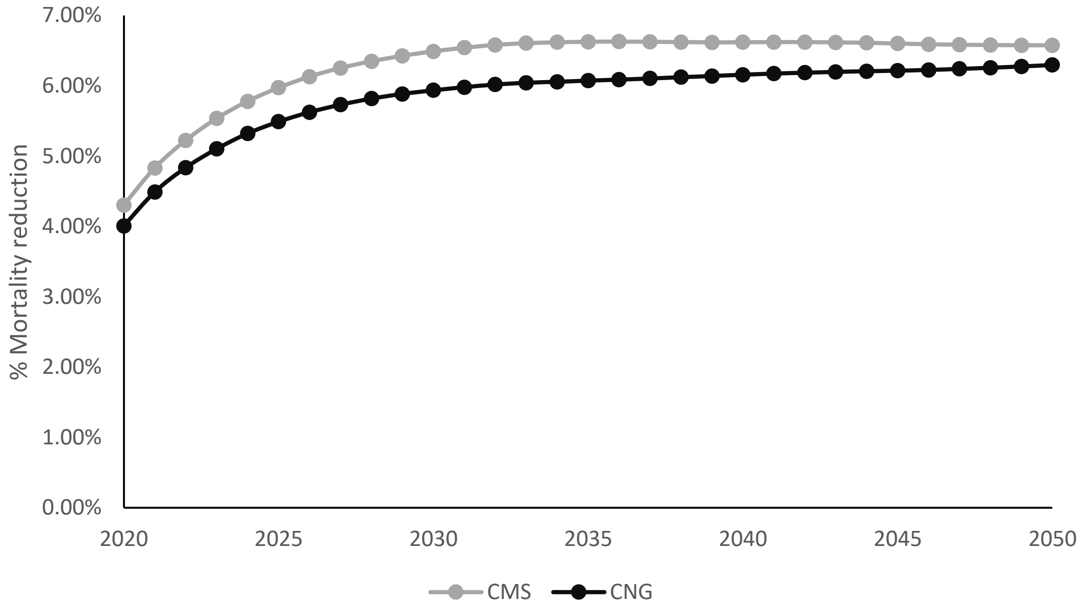

B)

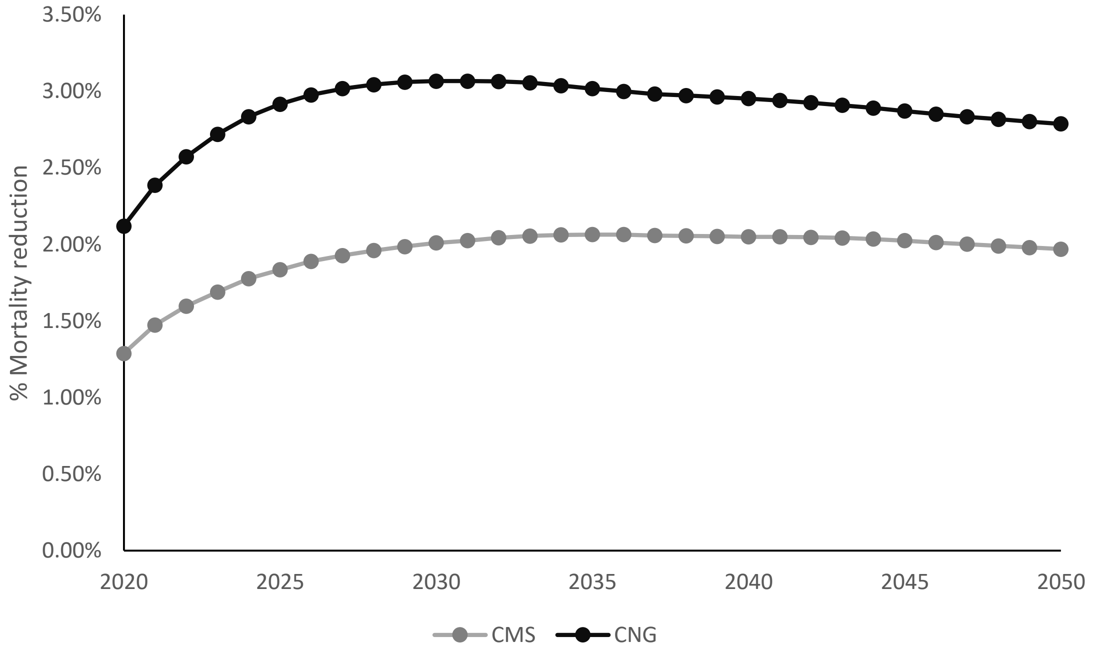

Supplement: S2 Fig — (PDF) [file pone.0173119.s005.pdf]
