# Supplementary figures and images for: Evaluating lung cancer screening in China: Implications for eligibility criteria design from a microsimulation modeling approach
Source: PLoS One. 2017 Mar 8;12(3):e0173119. doi: 10.1371/journal.pone.0173119 (PMC5342219; doi:10.1371/journal.pone.0173119)

**S3 Fig. Mortality reduction compared to National Lung Screening Trial (NLST) for the 1960 birth cohort.**

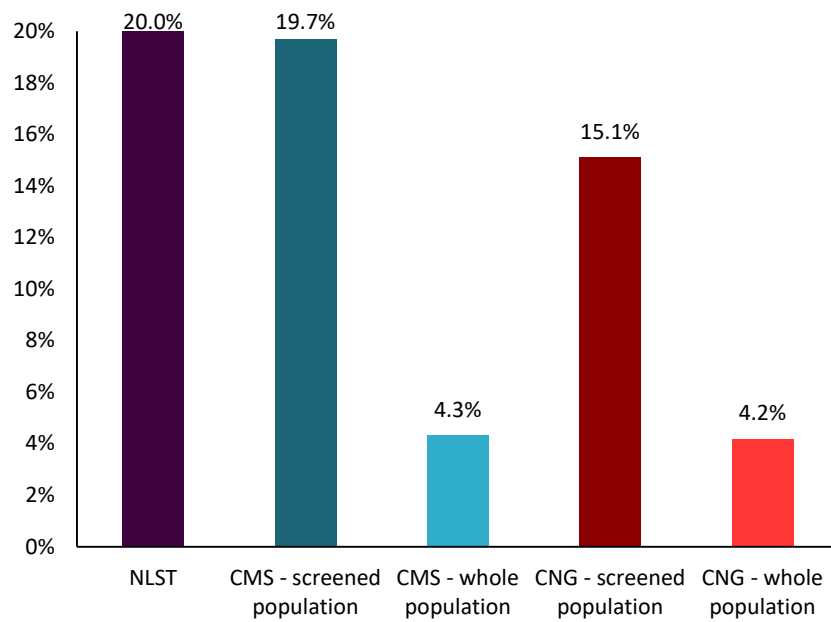

Supplement: S3 Fig — (PDF) [file pone.0173119.s006.pdf]
