# Supplementary material for: Evaluating lung cancer screening in China: Implications for eligibility criteria design from a microsimulation modeling approach
Source: PLoS One. 2017 Mar 8;12(3):e0173119. doi: 10.1371/journal.pone.0173119 (PMC5342219; doi:10.1371/journal.pone.0173119)

S4 Fig. Number of screening CT exams based on screening adherence rates to CMS strategy, 2016-2050. A) Males B) Females.

A)

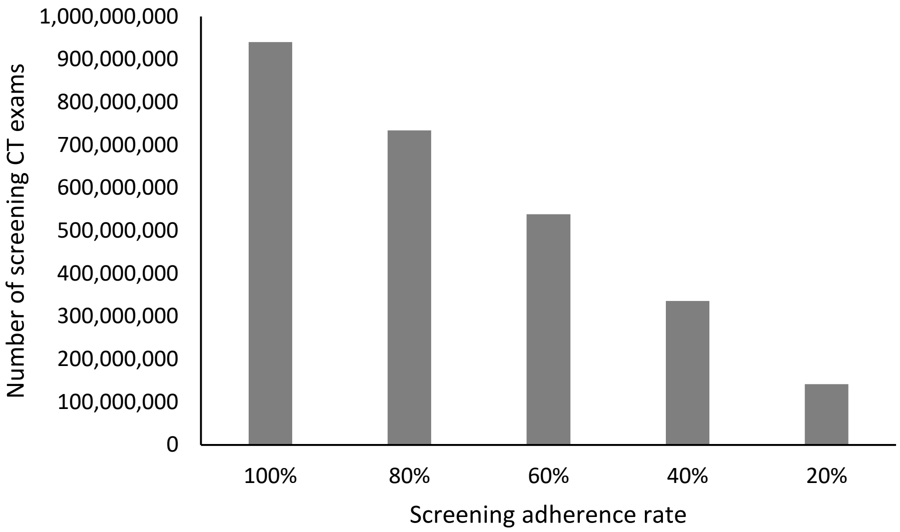

B)

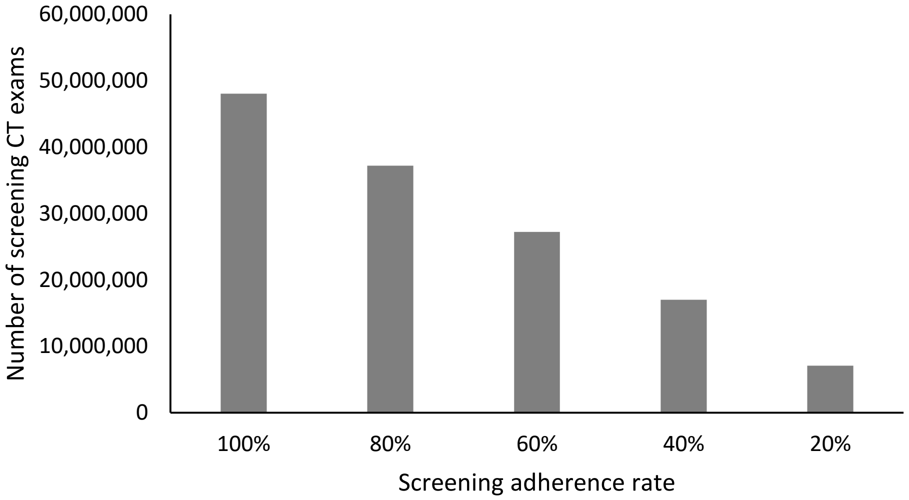

Supplement: S4 Fig — (PDF) [file pone.0173119.s007.pdf]
