# Supplementary material for: Evaluating lung cancer screening in China: Implications for eligibility criteria design from a microsimulation modeling approach
Source: PLoS One. 2017 Mar 8;12(3):e0173119. doi: 10.1371/journal.pone.0173119 (PMC5342219; doi:10.1371/journal.pone.0173119)

S5 Fig. Cigarettes per day. Inputs for cigarettes per day, by age (0-99) and year of birth, were adapted to values from the literature on smoking intensity in China (Chen 2015; Ng 2015; Qian 2010). In the paper by Chen et al., values for cigarettes per day are given for five different ages in the year 2006; these five values were extrapolated into a smooth curve for ages 20-100 (red) and our model was adapted to these values (gray).

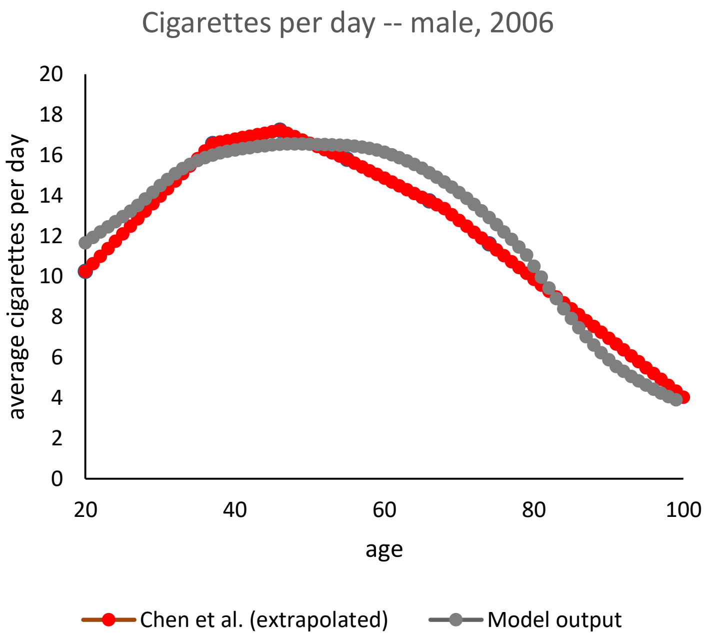

Supplement: S5 Fig — (PDF) [file pone.0173119.s008.pdf]
